# Active Contours Using Additive Local and Global Intensity Fitting Models for Intensity Inhomogeneous Image Segmentation

**DOI:** 10.1155/2016/9675249

**Published:** 2016-10-09

**Authors:** Shafiullah Soomro, Farhan Akram, Jeong Heon Kim, Toufique Ahmed Soomro, Kwang Nam Choi

**Affiliations:** ^1^Department of Computer Science and Engineering, Chung-Ang University, Seoul 156-756, Republic of Korea; ^2^Department of Computer Engineering and Mathematics, Rovira i Virgili University, 43007 Tarragona, Spain; ^3^Korea Institute of Science and Technology Information, Daejeon 305-806, Republic of Korea; ^4^School of Computing and Mathematics, Charles Sturt University, Bathurst, NSW 2795, Australia

## Abstract

This paper introduces an improved region based active contour method with a level set formulation. The proposed energy functional integrates both local and global intensity fitting terms in an additive formulation. Local intensity fitting term influences local force to pull the contour and confine it to object boundaries. In turn, the global intensity fitting term drives the movement of contour at a distance from the object boundaries. The global intensity term is based on the global division algorithm, which can better capture intensity information of an image than Chan-Vese (CV) model. Both local and global terms are mutually assimilated to construct an energy function based on a level set formulation to segment images with intensity inhomogeneity. Experimental results show that the proposed method performs better both qualitatively and quantitatively compared to other state-of-the-art-methods.

## 1. Introduction

Image segmentation is a basic and important problem in the areas of computer vision, pattern recognition, and image processing. The presence of noise, low contrast, and intensity inhomogeneity affects the accuracy of intensity based image segmentation methods. Intensity inhomogeneity is a smooth varying intensity present in different parts of an intensity inhomogeneous image, which makes it difficult to separate object boundaries from the background. In medical image modalities, such as microscopy, computed tomography (CT), and magnetic resonance imaging (MRI), it manifests itself as a smooth intensity variation across the image during image acquisition process or because of outer interference. Numerous methods [1–10] have been devised to segment intensity inhomogeneous images. Active contour method proposed by Kass et al. is one of those methods, which is used to segment images with intensity inhomogeneity.

An active contour method segments an image by deforming a curve towards object boundary using a certain force under an energy minimization principle [11]. This technique was centered on few important characteristics like image gradient and statistical and curvature information. Active contour methods are further divided into two classes: edge based [11–17] and region based [1–10, 18–26] methods. Generally, both of these methods carry some advantages and disadvantage. Edge based methods deploy a force to deform a curve towards the object boundary of the given image by utilizing image gradient information. These methods experience the ill effects of energy leakage. Moreover, they do not perform well on images with low contrast, intense noise, and weak edges. In turn, region based methods yield better performance over noisy and blurred images. Traditional region based active contour methods work on an assumption that the given image is homogeneous [22, 27]. Therefore, they cannot properly segment images with intensity inhomogeneity. Among region based methods, only [20, 21] yield some capability to segment under intensity inhomogeneity these models widely known as piecewise smooth (PS) models. However, these methods involve some complex parameter setting, which makes them computationally expensive and impractical.

Li et al. proposed a local binary fitted (LBF) model [1, 2] to target image segmentation under intensity inhomogeneity. It computes image local region information by using a Gaussian kernel. In most cases, this method can properly segment images in the presence of intensity inhomogeneity. However, this method is extremely sensitive to initialization, which limits its usage in different applications. Rajapakse and Kruggel developed a statistical method [3] for inhomogeneous image segmentation. This method exploits the spatial information of neighboring pixels in the same class by incorporating Markov random fields (MRF) in formulation. Chen et al. proposed a method [4] as a variant of Mumford-Shah [21] model for inhomogeneous image segmentation. This method uses a Gaussian kernel based bias field estimator to handle intensity inhomogeneity. Its variational formulation properly segments the given image and accurately finds the global minimum of energy functional. However, its energy functional is constructed with an assumption that intensity variations are represented by linearly combined *K* characteristic functions, each identifying one segment region. Li et al. proposed an intensity inhomogeneous image segmentation method [5] based on LBF method known as multiplicative image segmentation model for intensity inhomogeneity. It constructs an energy functional, which includes a bias field that models the smooth variations of intensity inhomogeneity. Mukherjee and Acton also contributed to image segmentation and proposed a method [6] which segments images with significant intensity variation. It models the foreground and background using Legendre basis functions in which region intensities are represented in lower dimensional space to permit smoothness.

Recently, Min et al. proposed a method [29] which uses a new global intensity term based on global division algorithm. This term boosts the performance of Chan-Vese [22] method and helps to capture the complicated image intensity information in a better way compared to Chan-Vese method. This paper presents a new region based active contour model for level set formulation with an additive formulation of energy functional using both local and global intensity fitting terms. Local intensity term is dominant near object boundaries and it compels the contour towards the object boundaries, whereas the global intensity term which incorporates global image information helps to properly segment homogeneous regions.

The rest of the paper is organized as follows. In Section 2, theoretical foundations are discussed.  Section 3 presents the proposed model. In Section 4, experimental results using different images are shown. Finally, discussion and conclusions are given in Sections 5 and 6.

## 2. Theoretical Foundations

### 2.1. Mumford-Shah Model

Chen et al. proposed a region based method [4] for image segmentation using a piecewise approximation. Their method aims to find an optimal piecewise approximation function *u* which approximates an image *I* smoothly within each subregion of the image domain *Ω*_*i*_ ⊂ *R* ^2^. They proposed the following energy functional:

(1) EMSu,C=λ∫ΩI−ux2dx+v∫Ω∖C∇u2dx+μC,

where |*C*| represents the length of the contour and *μ*, *v* ≥ 0 are fixed parameters. In (1), the first term is the fitting term, the second term is the smoothing term, and the third term regularizes the curve. The unknown contour *C* and the nonconvexity of the above energy functional make it difficult to minimize it. Numerous methods have been proposed by modifying the above energy functional, some of which are explained in the later part of this section.

### 2.2. Chan-Vese Model

Chan and Vese [22] proposed a region based active contour method based on Mumford and Shah [27]. They proposed the following energy functional for piecewise segmentation:

(2) EMSμ,c1,c2=λ1∫ΩIx−c12Hεϕxdx+λ2∫ΩIx−c221−Hεϕxdx+μLC+vC,

where *I*(*x*) is a given image; the first and second terms in (2) represent force terms using intensities both inside and outside contour *C*, respectively. *L*(*C*) is the length of the contour. *λ*_1_, *λ*_2_, *μ*, and *v* are fixed parameters. *c*_1_ and *c*_2_ are image intensity means inside and outside the region, respectively. By minimizing (2) with respect to *c*_1_, *c*_2_, and *ϕ* using the steepest gradient descent [30], the following definitions and solution equation are acquired:

(3) c1=∫ΩIxHεϕxdx∫ΩHεϕxdx,

(4) c2=∫ΩIx1−Hεϕxdx∫Ω1−Hεϕxdx,

(5) ∂ϕ∂t=−λ1I−c12+λ2I−c22+μ div⁡∇ϕ∇ϕ−vδεϕ,

where *H* is Heaviside function and *δ* is Dirac delta function. The constant *ε* controls the smoothness of Heaviside function and width of Dirac Delta function. The smoothed approximations of Dirac and Heaviside functions are defined as follows:

(6) Hεϕ=121+2πarctan⁡ϕε,

(7) δεϕ=επϕ2+ε2.

### 2.3. LBF Model

Li et al. proposed a local binary fitted (LBF) active contour method [1, 2], which yields local image intensity information to segment images with intensity inhomogeneity. The basic idea is to introduce a Gaussian kernel function to define the LBF energy functional as follows:

(8) ELBFC,f1,f2=λ1∫ΩKσx−yIy−f1x2·Hϵϕydy+λ2∫ΩKσx−y·Iy−f2x21−Hϵϕydy.

**Figure 1:**
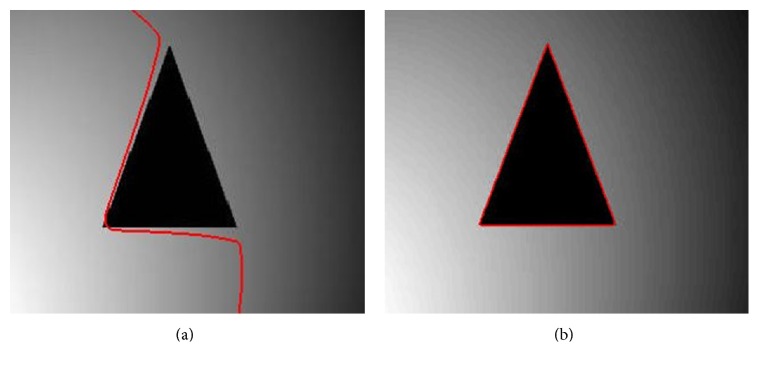
Intensity inhomogeneous image segmentation using global and local intensity based active contour methods. (a) Segmentation result using Chan-Vese method. (b) Segmentation result using LBF method.

Minimizing the energy formulation in (6) with respect to *f*_1_ and *f*_2_ using the steepest gradient descent [30], the following definitions are acquired:

(9) f1x=Kσ∗HϵϕIxKσ∗Hϵϕ,

(10) f2x=Kσ∗1−HϵϕIxKσ∗1−Hϵϕ,

where *K*_*σ*_ is the Gaussian kernel with standard deviation *σ*. *f*_1_ and *f*_2_ are local intensity means inside and outside the region. This method can segment inhomogeneous images by embedding the local information inside the energy formulation. It yields far better result than traditional region based active contour methods [22, 27].  Figure 1 shows image segmentation results using both traditional region based active contour (Chan-Vese) method [22] and LBF method [1, 2].  Figure 1(a) shows that the Chan-Vese method could not segment a homogeneous object from the inhomogeneous background. On the other hand, Figure 1(b) shows that the LBF method could properly segment a homogeneous object from the inhomogeneous background. In most cases, LBF method can segment images with intensity inhomogeneity; however, it is more sensitive to level set initialization. Localization property of this method develops too many local minimums; therefore, its segmentation accuracy is dependent on the initial position of level set.

### 2.4. Min et al.'s Model

**Figure 2:**
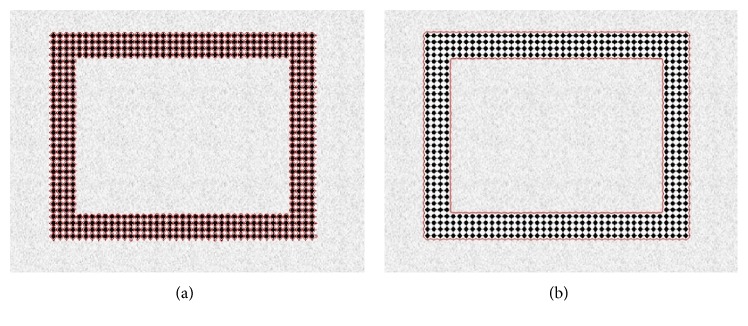
Segmentation of a complex (texture-like) region. (a) Segmentation result using Chan-Vese method. (b) Segmentation result using Min et al.'s method.

Chan-Vese model does not depend on image gradient as previous models do. Moreover, it can also properly segment noisy images under a certain level of noise. However, it is unable to segment complex intensity regions, which contain pixels of big intensity difference. In order to solve this problem, Min et al. proposed a method with an effective global region term which can capture complicated intensity regions. It formulates an energy functional based on global division algorithm and develops a novel region based term which can properly segment objects with big intensity difference or affected by noise. Their energy functional based on a novel region based term is defined as follows:

(11) EMinϕ,c1,c2,d11,d12,d21,d22=∫ΩHIx−c1·Ix−d112Hϵϕxdx+∫Ω1−HIx−c1Ix−d122·Hϵϕxdx+∫ΩHIx−c2Ix−d122·1−Hϵϕxdx+∫Ω1−HIx−c2·Ix−d2221−Hϵϕx,

where *H*(*I*(*x*) − *c*_*i*_)  (*i* = 1,2) is a division function based on intensity magnitude. Instead of computing one intensity mean inside the object and one outside, this method computes two values (bigger and smaller) of intensity means inside the object and two values of intensity means outside. By minimizing (11) with respect to *d*_11_, *d*_12_, *d*_21_, and *d*_22_ using the steepest gradient descent [30], the following definitions are acquired:

(12) d11=∫ΩHIx−c1IxHϵϕxdx∫ΩHIx−c1Hϵϕxdx,

(13) d12=∫Ω1−HIx−c1IxHϵϕxdx∫Ω1−HIx−c1Hϵϕxdx,

(14) d21=∫ΩHIx−c2Ix1−Hϵϕxdx∫ΩHIx−c21−Hϵϕxdx,

(15) d22=∫Ω1−HIx−c2Ix1−Hϵϕxdx∫Ω1−HIx−c21−Hϵϕxdx,

where *d*_11_ and *d*_12_ are bigger and smaller mean intensity values inside the contour. Similarly, *d*_21_ and *d*_22_ represent the bigger and smaller intensities outside the contour. Chan-Vese method cannot properly segment an object if it contains big intensity differences. By using two values of intensity means unlike Chan-Vese method, this method decreases the probability of error in the segmentation process.  Figure 2 shows segmentation of a complex (texture-like) region using Chan-Vese and Min et al.'s methods.  Figure 2(a) shows that Chan-Vese method yields unacceptable segmentation. It considered small black dots as individual regions and, therefore, could not properly segment the big rectangular region, which is the region of interest. On the other hand, Min et al.'s method properly segmented the rectangular region as shown in Figure 2(b).

## 3. The Proposed Method

The proposed method exploits the advantages of both LBF and Min et al.'s methods by incorporating their local and global intensity information. The proposed energy formulation is defined as
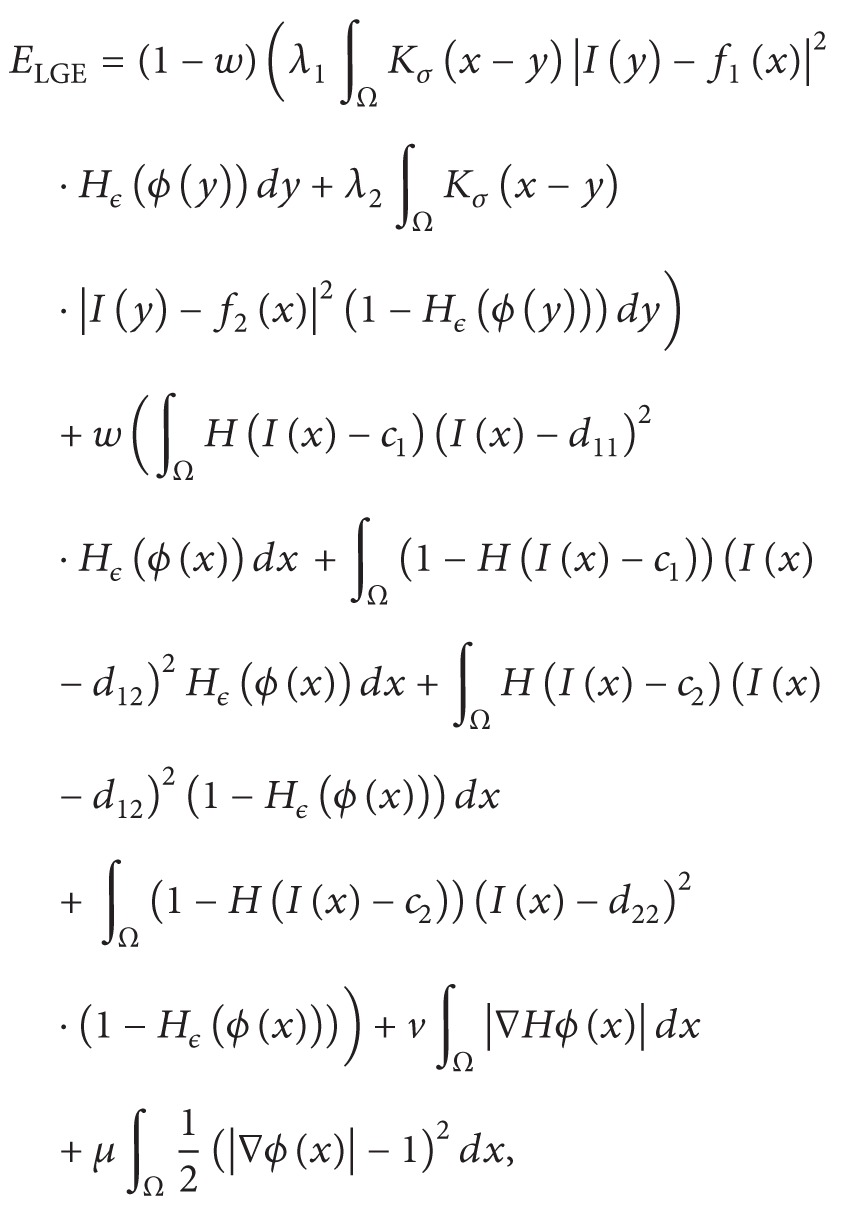
(16)where *w* is a positive constant 0 ≤ *w* ≤ 1, which plays an important role in handling intensity inhomogeneity during the segmentation process. When *w* is close to 0, then the proposed energy function has a dominant local force term and when it is close to 1 then it has a dominant global force term. *v*, *μ* > 0 are scale parameters for length term and energy penalization term, respectively. The second last term in (16) is length term which is used to regularize the curve. The last term is the energy penalizing term which maintains level set function as signed distance function (SDF). Moreover, it also removes the computationally expensive need of reinitialization.

By minimizing *E*_LGE_ in (16) with respect to *ϕ* using the steepest gradient descent [30], the following solution is obtained:

(17) ∂ϕ∂t=δϵϕF1+F2+vδϵϕdiv⁡∇ϕ∇ϕ+μ∇2ϕ−div⁡∇ϕ∇ϕ,

where *F*_1_ and *F*_2_ are local and global force terms, respectively. Local force term *F*_1_ is defined as

(18) F1=1−w−λ1∫ΩKσx−yIy−f1x2dy+λ2∫ΩKσx−yIy−f2x2dy,

where *f*_1_ and *f*_2_ are local intensity means inside and outside the region defined in (9) and (10), respectively. Global force term *F*_2_ is defined as

(19) F2=w−λ1HεI−c1I−d112−λ11−HεI−c1I−d122+λ2HεI−c2I−d212+λ21−HεI−c2I−d222,

where *c*_1_ and *c*_2_ are global intensity means inside and outside the region from Chan-Vese method [22], which are defined in (3) and (4), respectively. *d*_11_, *d*_12_, *d*_21_, and *d*_22_ are global intensity means (*d*_11_, *d*_12_) inside and (*d*_21_, *d*_22_) outside the region from Min et al.'s method [29], which are defined in (12), (13) and (14), (15), respectively. The terms *d*_11_, *d*_12_, *d*_21_, and *d*_22_ depend on the division function *H*(*I*(*x*) − *c*_*i*_)  (*i* = 1,2) which is based on intensity magnitude. The division function allows capturing the small changes in the intensity differences, which leads to better segmentation results compared to traditional global intensity based active contour methods. The proposed method employs smoothed versions of Heaviside function *H*(*ϕ*) and Dirac function *δ*(*ϕ*) as defined in (5) and (6), respectively.

**Figure 3:**
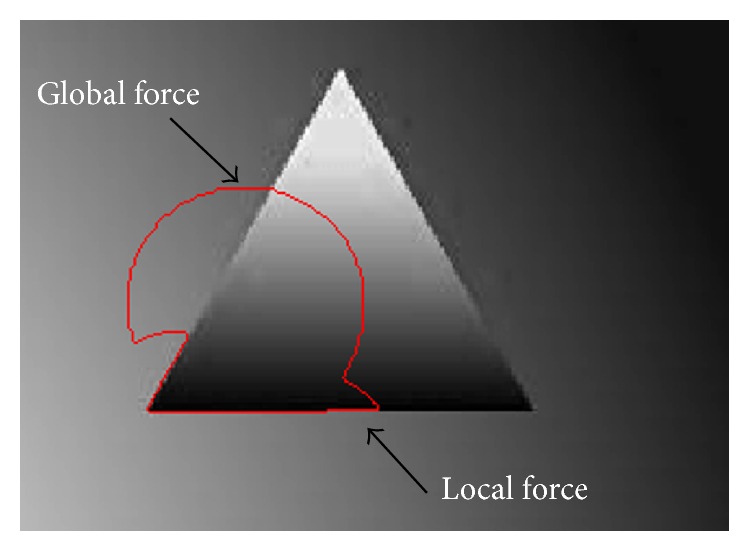
Effect of local and global intensity term on inhomogeneous image.

Both forces *F*_1_ and *F*_2_ play an important role during the curve evolution process. Because *F*_1_ force term is dependent on local intensity means, therefore, when it is dominant, that is, *w* is close to 0, then the contour moves towards the (intensity inhomogeneous) object as shown in Figure 3. On the other hand, when *F*_2_ force term, which is dependent on global intensity means, is dominant, then the contour moves away from the intensity inhomogeneous object as shown in Figure 3.

For outmoded level set techniques, it is essential to set initialization of *ϕ* as signed distance function (SDF), because when it is meaningfully not the same as SDF, then the reinitialization techniques are no longer competent to reinitialize the function as SDF. The proposed formulation eliminates computationally expensive reinitialization procedure by using the penalizing energy term from [16]. Initial level set function for the proposed method is defined here as

(20) $$\begin{array}{c} \phi \left(\;x,t=0\;\right)=\left\{\begin{array}{cc} -\rho & x\in \Omega_{0}-\partial \Omega_{0}\\ 0 & x\in \partial \Omega_{0}\\ \rho & x\in \Omega -\Omega_{0}, \end{array}\right. \end{array}$$

where *ρ* > 0 is constant (in this paper *ρ* = 2). Finally, the iterative steps of the proposed method are summarized as follows:

(1) Initialize level set function *ϕ* using *ϕ*_0_ from (20).
(2) Compute *f*_1_ and *f*_2_ using (9) and (10), respectively.
(3) Compute *c*_1_, *c*_2_, *d*_11_, *d*_12_, *d*_21_, and *d*_22_ using (3), (4), (12), (13), (14), and (15), respectively.
(4) Solve the partial differential equation (PDE) for *ϕ* with (17).
(5) Return to step (2) until convergence.

## 4. Experimental Analysis and Comparison

The proposed method is applied to various synthetic and medical images from different modalities. It is implemented using MATLAB 8.5 in Windows 8 environment on a 2.97 GHz Intel Core-i7 processor with 4 GB RAM. The parameters used for all methods in all experiments are shown in Table 1.

**Figure 4:**
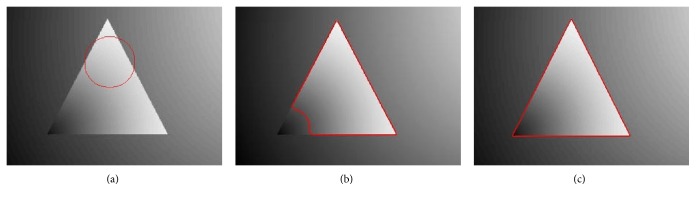
Intensity inhomogeneous image segmentation using synthetic image. (a) Image with initial contour. (b) Result using LBF method. (c) Result using the proposed method.

Figure 4 shows intensity inhomogeneous image segmentation using the proposed method and LBF method.  Figure 4(a) shows original image with initial contour.  Figure 4(b) shows that LBF method failed to properly segment intensity inhomogeneous object. In turn, the proposed method properly segmented intensity inhomogeneous object as shown in Figure 4(c).

Image segmentation under intensity inhomogeneity is a well-known problem in medical image analysis. The presence of intensity inhomogeneity can lead to high false positives when intensity based image analysis tools are used.

**Figure 5:**
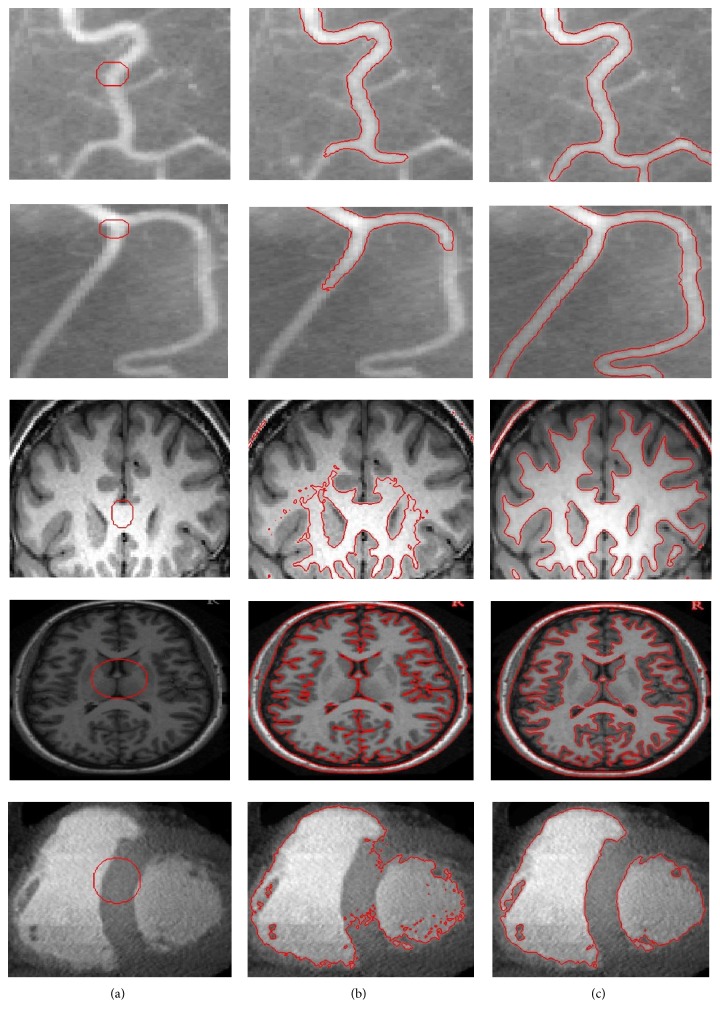
Curve evolution process from initial contour to final contour. Initial contour (a). Contour after 20 iterations (b). Final contour (c).

Figure 5 illustrates the segmentation process of five different medical images with intensity inhomogeneity. The first and second rows show contour evolution process from initial contour to final contour on vessel images. Final contour in the second and third rows shows that the proposed method properly segmented weak edged vessels in both images. The third and fourth rows show contour evolution process on brain MR images. Final contour in the third and fourth rows shows that the proposed method properly segmented white matter regions in both images. The last row shows contour evolution process of the proposed method on cardiac computed tomography (CT) image. It shows that the proposed method properly segmented the left ventricle (LV) and right ventricle (RV).

**Figure 6:**
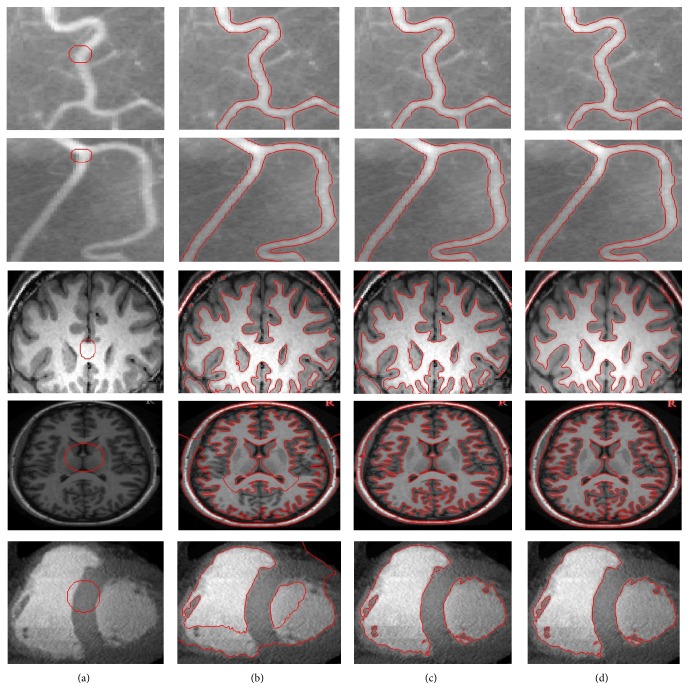
Image segmentation using medical images with intensity inhomogeneity. (a) Initial contour (first column). (b) Segmentation results using LBF method [2] (second column). (c) Segmentation results using Wang et al.'s method [28] (third column). (d) Segmentation results using the proposed method (last column).

Figure 6 shows a segmentation result comparison between the LBF [2], Wang et al.'s [28], and the proposed method using different medical images. It shows that both the proposed and Wang et al.'s methods properly segmented all images. Although Wang et al.'s method segmented regions of interest in all images, it missed some part of weak boundaries of vessels as shown in the first two rows. On the other hand, LBF method accurately segmented images in the first three columns but it failed to properly segment brain MRI image in the fourth column and cardiac CT image in the last column.

**Figure 7:**
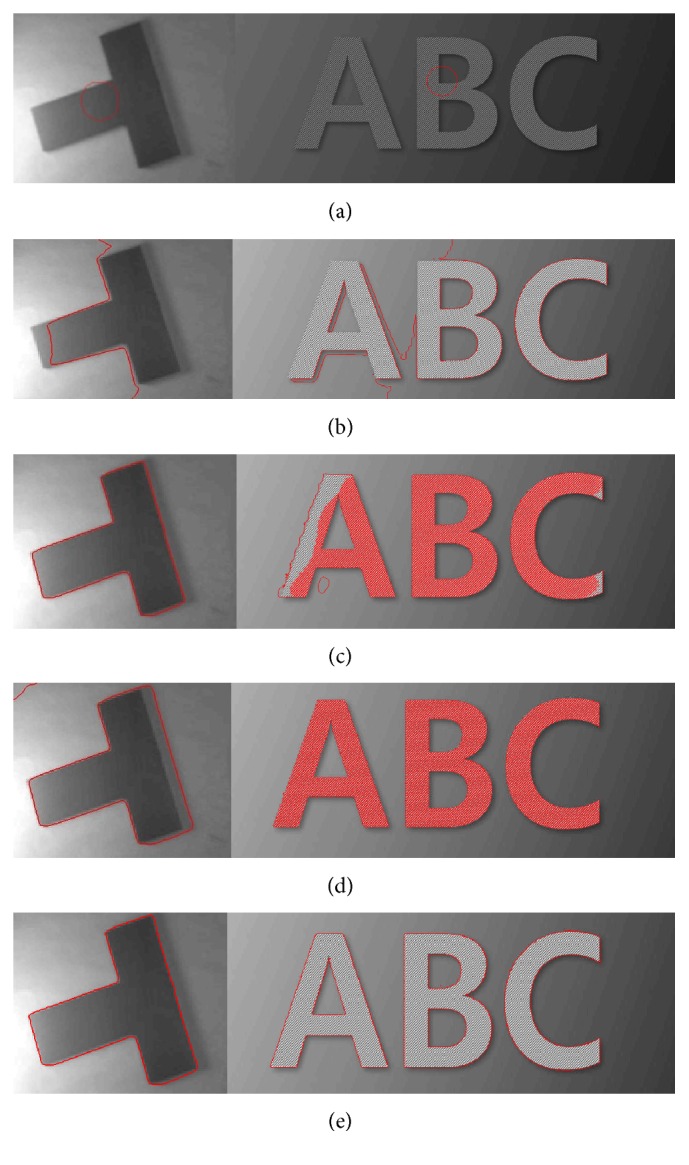
Image segmentation using synthetic images with intensity inhomogeneity. (a) Original images with initial contour (first row). (b) Segmentation result using Chan-Vese method [22] (second row). (c) Segmentation result using LBF method [2] (third row). (d) Segmentation result using Wang et al.'s method [28] (fourth row). (e) Segmentation result using the proposed method (last row) at *σ* = 3.0, *λ*_1_ = 1, *λ*_2_ = 2, and *w* = 0.1/10^8^ for image 1 and *w* = 0.1/10^9^ for image 2.

Figure 7 shows a segmentation result comparison between Chan-Vese [22], LBF [2], Wang et al.'s [28], and the proposed method using synthetic images with intensity inhomogeneity. In Figure 7, the first row shows original images with initial contour, the second row shows result using Chan-Vese method, the third row shows result using LBF method, the fourth row shows result using Wang et al.'s method, and the last row shows result using the proposed method. First images from Figures 7(c) and 7(e) show that both LBF and the proposed methods yield the best segmentation results for the first image in Figure 7. Moreover, Figure 7(e) also shows that the proposed method yields the best segmentation result for the second image. In turn, Chan-Vese method could not properly segment both images. Although Wang et al.'s method could segment the first image, it segmented the shadow region (unable to differentiate between background and objects shadow). In turn, both LBF and Wang et al.'s methods are unable to properly segment the second image.

**Figure 8:**
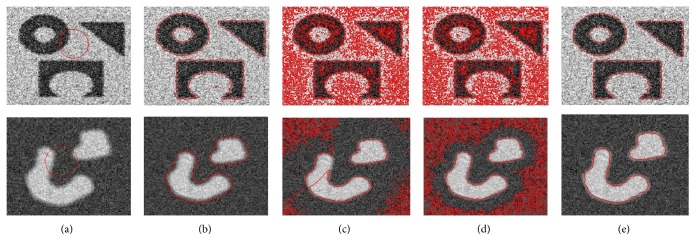
Image segmentation using noisy images. (a) Original images with initial contour (first column). (b) Segmentation result using Chan-Vese method [22] (second column). (c) Segmentation result using LBF method [2] (third column). (d) Segmentation result using Wang et al.'s method [28] (fourth column). (e) Segmentation result using the proposed method (fifth column) at *w* = 0.1 for image 1 and 0.001 for image 2.

Figure 8 shows segmentation result comparison between Chan-Vese [22], LBF [2], Wang et al.'s [28], and the proposed method using noisy images.

In this figure, the first column shows original images with initial contour, the second column shows segmentation result using Chan-Vese method, the third column shows segmentation result using LBF method, the fourth column shows segmentation result using Wang et al.'s method, and the last column shows segmentation result using the proposed method. The proposed method yields the best segmentation result for image shown in the first row. Chan-Vese method also properly segmented region of interest in that image but it also segmented some part of the noise. On the other hand, both the proposed and Chan-Vese methods yield the best segmentation result for the image shown in the second row. In turn, both LBF and Wang et al.'s methods could not segment images in both rows.

### 4.1. Quantitative Analysis

**Figure 9:**
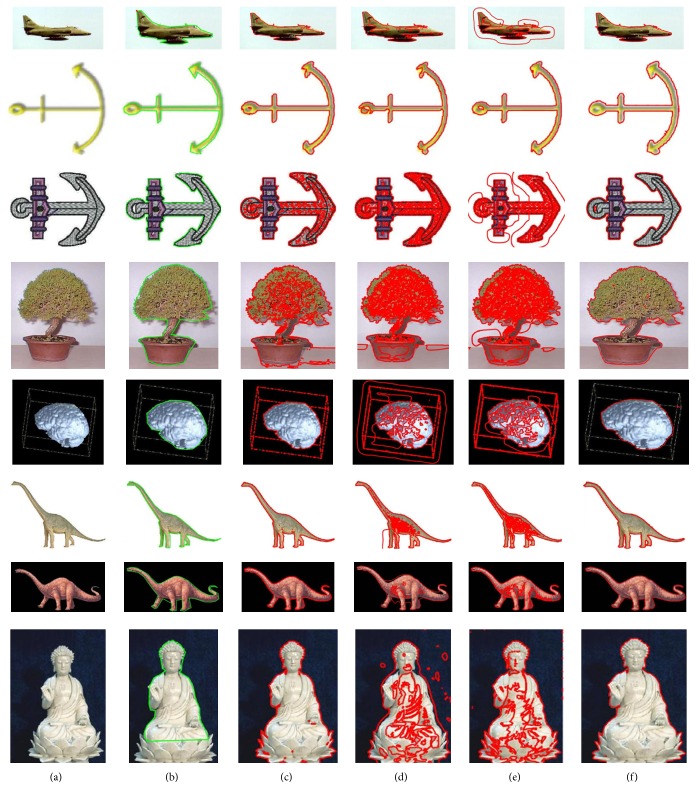
Image segmentation using real world images (a) Original image (first column). (b) Original images with ground truth (second column). (c) Segmentation result using Chan-Vese method [22] (third column). (d) Segmentation result using LBF method [2] (fourth column). (e) Segmentation result using Wang et al.'s method [28] (fifth column). (f) Segmentation result using the proposed method (sixth column).

**Figure 10:**
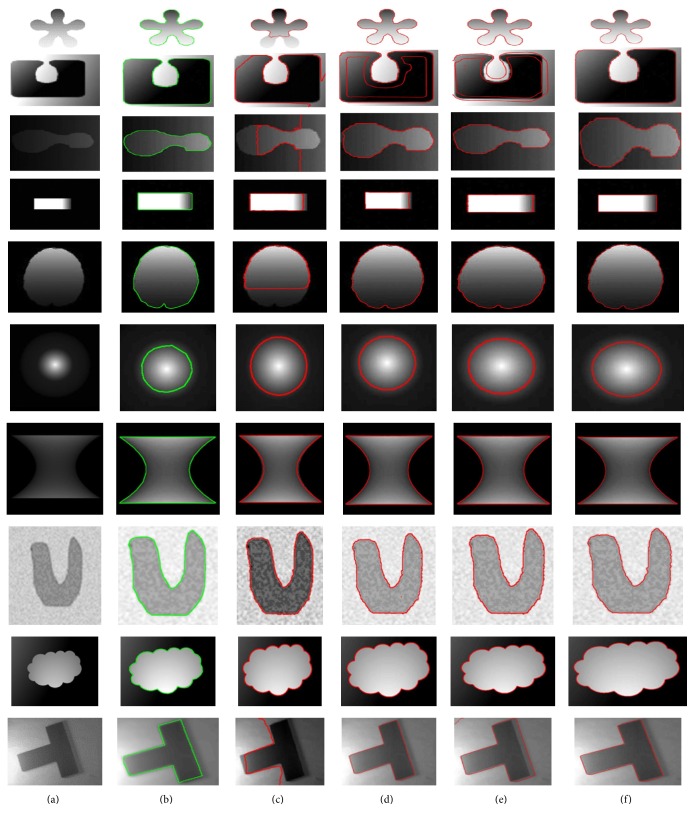
Image segmentation using synthetic images. (a) Original image (first column). (b) Original images with ground truth (second column). (c) Segmentation result using Chan-Vese method [22] (third column). (d) Segmentation result using LBF method [2] (fourth column). (e) Segmentation result using Wang et al.'s method [28] (fifth column). (f) Segmentation result using the proposed method (sixth column).

In this subsection, the results of the proposed method are quantitatively analyzed and compared with the state-of-the-art methods using both synthetic and real images. Real images and their ground truths were obtained from the online accessible database known as Caltech database [31]. In turn, the ground truths of synthetic images were obtained by using manual segmentation tool.  Figure 9 shows segmentation result comparison of the proposed method with Chan-Vese [22], LBF [2], and Wang et al.'s [28] method using real images. Original images and their ground truths are shown in the first and second columns, respectively. The segmentation results of Chan-Vese, LBF, Wang et al.'s, and the proposed methods are shown in the third, fourth, fifth, and sixth columns, respectively. Similarly, Figure 10 shows the segmentation result comparison of the proposed method with Chan-Vese, LBF, and Wang et al.'s methods using synthetic images, where original images and their ground truths are shown in the first and second columns, respectively. In turn, Chan-Vese, LBF, Wang et al.'s, and the proposed methods results are shown in the third, fourth, fifth, and last column, respectively.

In order to quantitatively evaluate and compare the proposed method with the other state-of-the-art methods, Dice coefficient (DSC) and Hausdorff distance (HD) metrics are being used, where Dice coefficient metric measures how well segmentation *S* overlaps the ground truth *G*. Segmentation results are considered the best when Dice value is close to 1. Dice coefficient metric is defined as

(21) $$\begin{array}{c} \mathrm{DSC}\left(\;G,S\;\right)=\frac{2\left|\Omega_{G}\cap \Omega_{S}\right|}{\left|\Omega_{G}\right|+\left|\Omega_{S}\right|}. \end{array}$$

HD measures the distance between segmented contour *S* and ground truth contour *G*. Segmentation method yields the best segmentation result when HD value is close to 0. HD metric is defined as

(22) HDG,S=max⁡maxi⁡dgi,S,maxj⁡dsj,G,

where *G* and *S* contours comprise set of points *G* = {*g*_1_, *g*_2_, *g*_3_,…, *g*_*n*_}, *S* = {*s*_1_, *s*_2_, *s*_3_,…, *s*_*n*_}, respectively, and *d* is the distance from *g*_*i*_ to the closest point on curve *S*.

**Figure 11:**
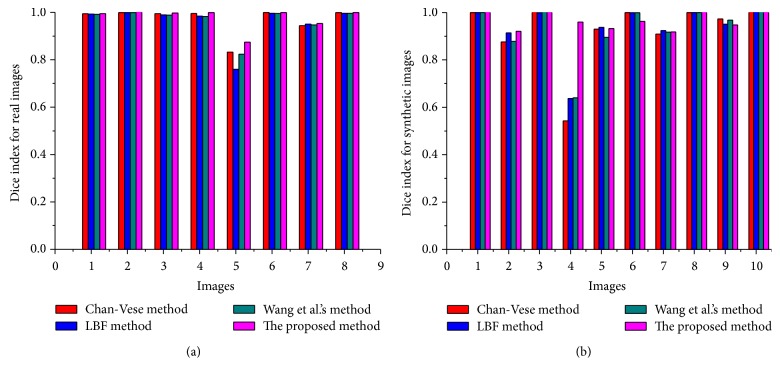
DSC values for set of real images (a) and synthetic images (b).

**Figure 12:**
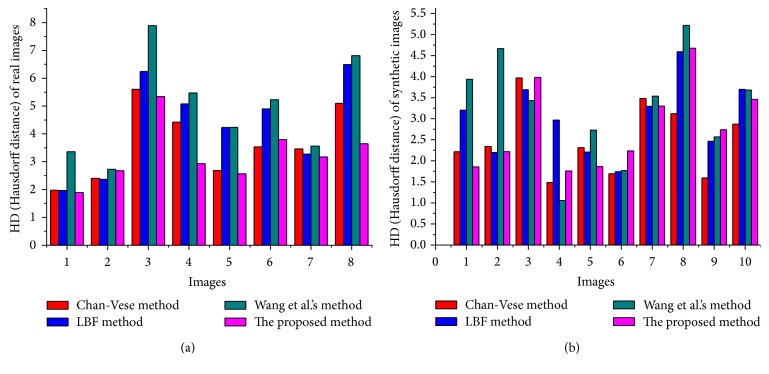
HD (Hausdorff distance) values for set of real images (a) and synthetic images (b).

**Figure 13:**
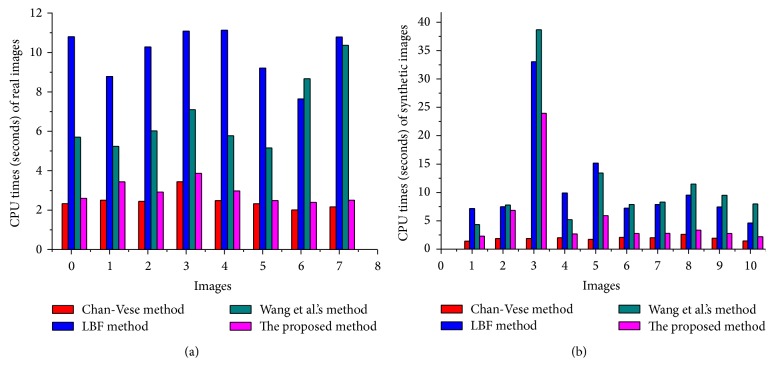
Comparison of our method in terms of CPU time (in seconds), real world images (a), and synthetic images (b).

In Figure 11, Dice index (DSC) values, using real and synthetic images from Figures 9 and 10, are shown. The proposed method yields the highest DSC values compared to the state-of-the-art methods. Similarly, Figure 12 shows Hausdorff distance (HD) values using real and synthetic images from Figures 9 and 10. It shows that the proposed method yields minimum HD values compared to other methods.  Figure 13 shows CPU time (in seconds) of each method using both real and synthetic images from Figures 9 and 10, respectively. It shows that the proposed method yields small CPU time compared to LBF [2] and Wang et al.'s [28] methods. Although Chan-Vese [22] method yields the smallest CPU time, it generates unacceptable segmentation results. On the other hand, the proposed method yields the best segmentation result with a little more CPU time compared to Chan-Vese method.

## 5. Discussion

### 5.1. The Parameter *w*

Constant parameter *w* in the proposed energy functional plays a crucial role in the segmentation process. This parameter manages the amount of local and global intensity force depending on the type of the image. In the proposed energy function, local intensity fitted term is scaled with a (1 − *w*) parameter and global intensity fitted term is scaled with *w* parameter, where 0 ≤ *w* ≤ 1. When input image has high level of intensity inhomogeneity, the value chosen for *w* should be close to 0 to reduce the interference of global fitted term. At *w* ≈ 0 local force term *F*_1_ is dominant and plays major role in segmenting intensity inhomogeneous objects because at that time global force term is close to zero; that is, *F*_2_ ≈ 0.

Similarly, if the image is homogeneous or is influenced by noise, then *w* should be close to 1 to make global force term *F*_2_ dominant. In case of noisy image, if *w* is not close to 1, then local force term *F*_1_ will negatively affect the segmentation accuracy. It will consider noise speckles as important information and will end up segmenting noise that is undesirable. Moreover, Gaussian kernel which is used to compute the local intensity mean will also considerably increase the time complexity. Value of *w* should be close to 0 if the given image is intensity inhomogeneous. In turn, it should be close to 1 if the image is homogeneous or noisy.

### 5.2. Relation with Other Methods

The proposed energy functional is formulated using local and global intensity means from level set methods [2, 7]. The global part of the proposed energy functional is based on Min et al.'s [29] global intensity term. In turn, the local part of the proposed energy functional is taken from LBF method [2]. Min et al.'s intensity term is an advance version of Chan-Vese [22] energy function, which has the ability to capture more complicated intensity information than Chan-Vese method as shown in Figure 2.

Traditional piecewise constant (PC) models [22, 27] cannot properly segment intensity inhomogeneity because they are proposed with an assumption that the given image is homogeneous. Several methods [3–6] have been proposed to handle intensity inhomogeneity. Among these methods, LBF method [1, 2] has superiority in terms of capturing inhomogeneity across the image. Rajapakse and Kruggel's [3] method has high time complexity because it smoothes the given image to deal with intensity inhomogeneity. On the other hand, the proposed method does not require image smoothing before segmentation. Similarly, Chen at el. [4], Li et al. [5], and Mukherjee and Acton [6] also contributed to inhomogeneous image segmentation. These methods have shown better results over several images but they have high computational complexity. Li et al.'s [5] method developed modified level set framework for inhomogeneous image segmentation based on LBF model. It models bias field to correct intensity variations in the given image with clustering property, which increases computational burden and time complexities.

The proposed method utilizes simple and robust formulation, which increases segmentation accuracy as shown in Figure 7(b). It has accurately segmented desired regions of interest in the presence of intensity inhomogeneity, while the rest of the methods failed to do so.

### 5.3. Limitation

The main limitation of the proposed method is high time complexity. Its energy functional has incorporated both local and global intensity means; therefore, it requires more time to process as compared to global intensity based methods. A possible solution is to develop a robust energy functional, which evolves faster on smooth homogeneous and inhomogeneous regions. The main idea is to use a phase shift in the Heaviside function as used in [32].

## 6. Conclusion

In this paper, a new region based active contour method with variational level set formulation is proposed. The proposed energy functional incorporates both local and global intensity terms in an additive way. Local intensity fitting term utilizes local information from the image, which helps to segment intensity inhomogeneous objects. In turn, global intensity fitting term helps to segment homogeneous regions. Chan-Vese method employs one intensity representative for inside and one for outside the contour. Therefore, it cannot properly segment complex homogeneous regions which have high intensity differences among the contained pixels.

On the other hand, the global intensity fitting term used in the proposed energy functional assimilates two intensity representatives for inside and two for outside the contour. Therefore, it can properly segment complex homogeneous regions. Moreover, an energy penalization term is used in level set formulation, which removes the need of reinitialization.

The proposed method is capable of segmenting images with intensity inhomogeneity with suitable initialization approach. Experimental results using several synthetic and medical images with intensity inhomogeneity demonstrate that the proposed method yields better segmentation results compared to the discussed state-of-the-art methods. Dice index, Hausdorff distance (HD), and CPU time comparison from Figures 11, 12, and 13 showed that the proposed method yields better results compared to the state-of-the-art methods. Although Chan-Vese method yields the smallest CPU time, it cannot properly segment images in Figures 9 and 10.

## Figures and Tables

**Table 1:** Parameter selection for all methods.

Parameters	The proposed method	LBF method [2]	Wang et al.'s method [28]
*σ*	5.0	3.0	3.0
*λ*_1_ = *λ*_2_	1	1	1.0
Time step	0.1	0.1	0.1
*μ*	1.0	1.0	1.0
*v*	0.004 × 255 × 255	0.001 × 255 × 255	0.001 × 25 × 255
*w*	0.1/10^8^	—	0.01
